# The epidemiology of pediatric outpatient acute respiratory tract infections in the US: a multi-facility analysis of multiplex PCR testing from 2018 to 2023

**DOI:** 10.1128/spectrum.03423-23

**Published:** 2023-12-14

**Authors:** Tristan T. Timbrook, Margaret Glancey, Brooklyn A. Noble, Stephen Eng, Zoe Heins, Benjamin Hommel, Marie Tessonneau, Ben W. Galvin, Grace Macalino

**Affiliations:** 1 Global Medical Affairs, bioMérieux, Salt Lake City, Utah, USA; 2 Department of Pharmacotherapy, College of Pharmacy, University of Utah, Salt Lake City, Utah, USA; 3 Baker Tilly US, LLP, Philadelphia, Pennsylvania, USA; 4 Data Science, bioMérieux, Salt Lake City, Utah, USA; 5 Baker Tilly US, LLP, New York, New York, USA; 6 Global Medical Affairs, bioMérieux, Marcy l'Étoile, France; 7 Baker Tilly, Tysons Corner, Virginia, USA; Johns Hopkins Hospital, Baltimore, Maryland, USA

**Keywords:** pediatric respiratory infections, multiplex PCR testing, pathogen prevalence, SARS-CoV-2, antibiotic stewardship, outpatient epidemiology

## Abstract

Acute respiratory tract infections in children increase the healthcare burden. Gaps exist in our understanding of the outpatient epidemiology of pediatric respiratory illnesses, especially following the emergence of SARS-CoV-2. The utilization of multiplex PCR testing can enhance our comprehension of these illnesses’ etiology. This study seeks to describe this epidemiology using these diagnostics. This study analyzed respiratory pathogen positivity from multiplex PCR testing between 2018 and 2023. Two multiplex PCR panels were utilized, with 15 viruses and four bacteria on one panel and 11 viruses and three bacteria on the other panel. The endpoints included pathogen rates, coinfection rates, and seasonal variations. Out of 38,778 tests from 16 pediatric sites, 75.3% occurred during 2021–2022. At least one pathogen was detected in 51.9%–67.8% of all tests annually, with human rhinovirus/enterovirus being the most common (27.2%–39.6% annual positivity). Despite a substantial increase in the number of tests per day during the COVID-19 pandemic (5.6 tests performed per day pre-pandemic vs 31.0 tests per day during the pandemic), the pre-pandemic period had similar overall pathogen (66.9% vs 61.4%) and viral (65.2% vs 61.3%) detection rates. This research emphasizes the value of multiplex PCR testing in elucidating the epidemiology of pediatric respiratory diseases between 2018 and 2023. Between 51.9% and 67.8% of annual results detected at least one pathogen, the majority viral. This underscores the significant potential for using data-driven approaches in antimicrobial stewardship to reduce unnecessary antibiotic use. Seasonal fluctuations of non-SARS-CoV-2 viruses suggest a shift toward the epidemiological patterns observed in the pre-pandemic era.

**IMPORTANCE:**

Post-pandemic, it is essential to understand the epidemiology of pediatric acute respiratory tract infections (ARTIs). Our multi-facility study elucidates the outpatient epidemiology of pediatric ARTI using highly multiplexed PCR testing, providing critical insights into the evolving landscape of the etiological agents with a particular focus on the years following the emergence of SARS-CoV-2. Utilizing data from two different multiplex PCR panels, our research provides a comprehensive analysis of respiratory pathogen positivity from 2018 to 2023. Our findings indicate that over half of the annual test results identified at least one pathogen, primarily of viral origin. Intriguingly, despite the surge in testing during the COVID-19 pandemic, pathogen detection rates remain similar to the pre-pandemic era. These data hold significant implications for directing antimicrobial stewardship strategies, curbing unnecessary antibiotic use in pediatric respiratory diseases, and the value of multiplex PCR testing in the outpatient setting among pediatrics.

## INTRODUCTION

Acute respiratory tract infections (ARTIs) in pediatrics are associated with significant healthcare burden, clinical consequences, and healthcare costs. ARTIs are estimated to account for at least 300 outpatient visits per 1,000 persons per year among children and adolescents in the United States (1). At least one-third of outpatient antibiotics prescribed for pediatrics are inappropriate, which can increase the risk of medication-induced adverse effects including gastrointestinal distress, *Clostridioides difficile*-associated diarrhea, and severe allergic reactions (2, 3). Inappropriate prescribing of antimicrobials has been associated with high 30-day attributable costs (3). Annual national costs for inappropriate antimicrobial use among pediatric outpatient respiratory presentations have been estimated to be $21.3 million for pharyngitis and $19.1 million for upper respiratory tract infections. Increased understanding of outpatient epidemiology in pediatric respiratory illness etiology will benefit antimicrobial stewardship interventions in this setting to improve patient outcomes and decrease antimicrobial resistance (AMR).

Knowledge of the epidemiology of outpatient pediatric respiratory illness is limited, particularly data after the onset of the circulation of SARS-CoV-2, and is likely different between healthcare settings (4 – 6). Moreover, general deficits in knowledge of viral epidemiology have been noted such as the Centers for Disease Control and Prevention (CDC) indicating gaps in epidemiology for respiratory syncytial virus (RSV) (7). The CDC’s National Respiratory and Enteric Virus Surveillance System (NREVSS) allows for insights into several viruses (respiratory syncytial virus , human parainfluenza viruses, human metapneumovirus, respiratory adenoviruses, human coronavirus, rotavirus, and norovirus) (8). However, NREVSS does not differentiate outpatient epidemiology and is limited by which viruses are captured (8). Expanded up-to-date etiological evaluations restricted to outpatient epidemiological data are needed.

The data from Clinical Laboratory Improvement Amendments of 1988 (CLIA)-waived multiplex PCR testing performed during routine clinical care have the potential to increase our understanding of the epidemiology of outpatient pediatric respiratory illness. This study aimed to describe the epidemiology of viral and atypical bacterial detections collected from a convenience sample of pediatric office-based practices in the United States utilizing the CLIA-waived RP1.7-EZ and RP2.1-EZ products before and during the COVID-19 pandemic from 2018 to 2023.

## MATERIALS AND METHODS

### Study design

This analysis described respiratory pathogen positivity and coinfections from the RP1.7-EZ panel from 22 January 2018 to 17 September 2021 and the RP2.1-EZ panel from 11 November 2020 to 24 March 2023 in an outpatient setting before (22 January 2018–10 March 2020) and after the start of the COVID-19 pandemic (11 March 2020–24 March 2023), using pandemic dates as defined by the World Health Organization (WHO) (9). All tests were collected as part of routine standard-of-care practice.

### Diagnostic tests

The CLIA-waived BIOFIRE FILMARRAY Respiratory 1.7-EZ (RP1.7-EZ) Panel and the BIOFIRE Respiratory 2.1-EZ (RP2.1-EZ) Panel, developed by BioFire Diagnostic, LLC were used. These tests simultaneously identify nucleic acids from various viruses and bacteria associated with respiratory tract infections from a single nasopharyngeal swab specimen. Acute respiratory infection pathogens tested on the RP1.7-EZ panel include 11 viruses [adenovirus, coronavirus (CoV-229E, CoV-HKU1, CoV-NL63, and CoV-OC43), human metapneumovirus, human rhinovirus/enterovirus, influenza A (subtypes H1, H3, and H1-2009), influenza B, parainfluenza virus (includes types 1–4), respiratory syncytial virus] and three bacteria (*Bordetella pertussis, Chlamydia pneumoniae*, and *Mycoplasma pneumoniae*). Similarly, acute respiratory infection pathogens observed on the RP2.1 EZ panel include 15 viruses [adenovirus, coronavirus (CoV-229E, CoV-HKU1, CoV-NL63, and CoV-OC43), coronavirus SARS-CoV-2, human metapneumovirus, human rhinovirus/enterovirus, influenza A (subtypes H1, H3, and H1-2009), influenza B, parainfluenza virus (includes types 1–4), respiratory syncytial virus] and four bacteria (*Bordetella parapertussis, Bordetella pertussis, Chlamydia pneumoniae*, and *Mycoplasma pneumoniae*). All test results that were collected on-site by clinic personnel between the time frames noted were included. Only de-identified data as defined by the Health Insurance Portability and Accountability Act of 1996 standards were received and used in the analyses. As the study used a fully de-identified database, it was exempt from ethics review under US 45 CFR 46.101(b)4.13.

### Study sites

The study included a convenience sample of all RP1.7-EZ and RP2.1-EZ test results performed from participating sites’ BioFire RP-EZ machines during the study period. Test results from a total of 16 office-based pediatric practices in the United States were included. Among available pediatric clinics, sites were chosen based on their utilization of RP-EZ, prioritizing sites with the highest sample volumes and the longest duration of machine use. All RP1.7-EZ and RP2.1-EZ tests performed at selected pediatric facilities that contributed data from 2018 to 2023 were included.

### Data analysis

Each test included facility name, facility type, zip code, and date. Tests were only included if they were complete and met control requirements. Tests were excluded when they were suspected of being quality control tests (positive for more than three pathogens) (10). No additional patient information was available for analysis.

Seasonality was defined as winter (1 December–28 February), spring (1 March–31 May), summer (1 June–31 August), and fall (1 September–30 November).

Primary endpoints included respiratory pathogen rates (annual, seasonal, pre-pandemic/pandemic, and by population density) and respiratory coinfection rates. All descriptive and statistical analyses were done using Python 3.10.

## RESULTS

The analysis comprised 38,778 unique test results from 16 pediatric office locations over the span of 5 years (2018–2023) (Table S1). The temporal distribution of tests was higher in the later years, with the majority of tests conducted during 2021 (14,544 tests) and 2022 (14,671 tests) (Table 1). Furthermore, the RP2.1-EZ panel was used considerably more (32,167 tests)than the RP1.7-EZ (6,611 tests).

**TABLE 1:** Overall and organism-specific detections by year

	2018	2019	2020	2021	2022	2023
Total tests (% total tests)	672 (1.7)	2,658 (6.9)	4,134 (10.7)	14,544 (37.5)	14,671 (37.8)	2,099 (5.4)
Any pathogen (% positivity)	439 (65.3)	1,792 (67.4)	2,147 (51.9)	8,793 (60.5)	9,454 (64.4)	1,423 (67.8)
Any viral pathogen	434 (64.6%)	1,741 (65.5%)	2,117 (51.2%)	8,792 (60.5%)	9,446 (64.4%)	1,421 (67.7%)
Adenovirus	35 (5.2%)	188 (7.1%)	170 (4.1%)	562 (3.9%)	842 (5.7%)	266 (12.7%)
Coronavirus	28 (4.2%)	134 (5.0%)	151 (3.7%)	612 (4.2%)	960 (6.5%)	244 (11.6%)
Coronavirus SARS-CoV-2^a^			93 (9.1%)	994 (6.9%)	1,676 (11.4%)	75 (3.6%)
Human metapneumovirus	15 (2.2%)	118 (4.4%)	105 (2.5%)	470 (3.2%)	514 (3.5%)	287 (13.7%)
Human rhinovirus/enterovirus	266 (39.6%)	864 (32.5%)	1,411 (34.1%)	5,290 (36.4%)	3,986 (27.2%)	663 (31.6%)
Influenza A	11 (1.6%)	113 (4.3%)	113 (2.7%)	84 (0.6%)	1,047 (7.1%)	42 (2.0%)
Influenza B	13 (1.9%)	40 (1.5%)	95 (2.3%)	67 (0.5%)	53 (0.4%)	4 (0.2%)
Parainfluenza virus	62 (9.2%)	323 (12.2%)	45 (1.1%)	1,169 (8.0%)	1,074 (7.3%)	114 (5.4%)
Respiratory syncytial virus	60 (8.9%)	198 (7.4%)	141 (3.4%)	1,047 (7.2%)	1,072 (7.3%)	56 (2.7%)
Any bacterial pathogen	5 (0.7%)	64 (2.4%)	42 (1.0%)	10 (0.1%)	13 (0.09%)	14 (0.7%)
*Bordetella parapertussis^a^*			0 (0.0%)	2 (0.0%)	6 (0.04%)	14 (0.7%)
*Bordetella pertussis*	0 (0.0%)	4 (0.2%)	6 (0.1%)	6 (0.04%)	3 (0.02%)	0 (0.0%)
*Chlamydia pneumoniae*	2 (0.3%)	14 (0.5%)	9 (0.2%)	1 (0.01%)	1 (0.01%)	0 (0.0%)
*Mycoplasma pneumoniae*	3 (0.4%)	46 (1.7%)	27 (0.7%)	1 (0.01%)	3 (0.02%)	0 (0.0%)

^a^ RP2.1-EZ test only.

Results on the primary analyses of overall and organism-specific detection counts and rates by year can be found in Table 1. In a given year, testing with detection of any pathogen ranged from 64.4% to 67.8%, except for in 2020 and 2021, which dropped to 51.9% and 60.5% positivity, respectively. Viral detection rates closely mirrored these proportions, as atypical bacteria made up 1% or less of detections per year with the exception of 2019, which saw *Mycoplasma pneumoniae* detections rise to 1.7%, resulting in an overall detection rate of 2.4% for atypical bacteria. The *Mycoplasma pneumoniae* cases in 2019 clustered in Maryland (21), while 10 were from Alabama, 9 were from California, 4 were from Louisiana, 1 was from Texas, and 1 was from Connecticut.

Among specific viruses detected, human rhinovirus/enterovirus was the most common with a positivity rate of 27.2% to 39.6% per year. The SARS-CoV-2 strain of coronavirus was detected in 9.1% of tests in 2020 and 11.4% of tests in 2022 before decreasing to a 3.6% positivity rate in 2023. Among all positive tests, single pathogen detections occurred most often (84.2% of tests), followed by tests indicating two or three pathogen coinfections (15.8% of tests).

A sensitivity analysis was conducted to determine the impact of one site that represented 43% of the test results. Inclusion of this high population density site in Los Angeles significantly lowered overall viral pathogen positivity from 2021 to 2023 driven by lower positivity rates of adenovirus, coronavirus, human rhinovirus/enterovirus, influenza A, and Parainfluenza.

Test utilization increased moderately during the pandemic period, accounting for 88.7% of the total number of tests compared to just 11.3% performed before the pandemic (Table S2). Overall detection rate (66.9) and viral detection rate (65.2) were both higher in the pre-pandemic period compared to during-pandemic rates (61.4 and 61.3, respectively). The detection rates for adenovirus, coronavirus (non-SARS-CoV-2), human metapneumovirus, influenza A and B, parainfluenza virus, and respiratory syncytial virus all decreased during the pandemic, with the most notable decrease observed for influenza B, from 2.9% before to 0.4% during the pandemic. The detection rate of human rhinovirus/enterovirus remained relatively unchanged (31.5% and 32.3% before and during the pandemic, respectively).

**FIG 1:**
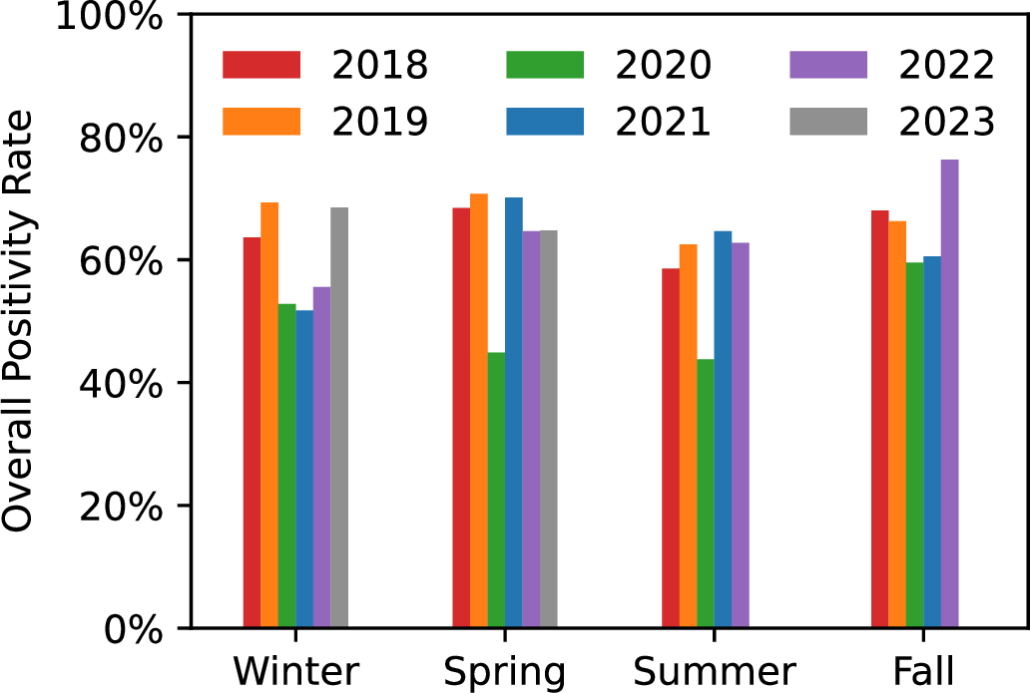
Pathogen positivity rate over study time 2018–2023. Graph begins in mid-2018 to censor weeks where no tests were conducted. Individual pathogen positivity at a given time point is represented as the difference between the highest and lowest positivity rate of the corresponding color. Total pathogen positivity at a given time point is represented as the peak pathogen positivity of all colors combined.

**FIG 2:**
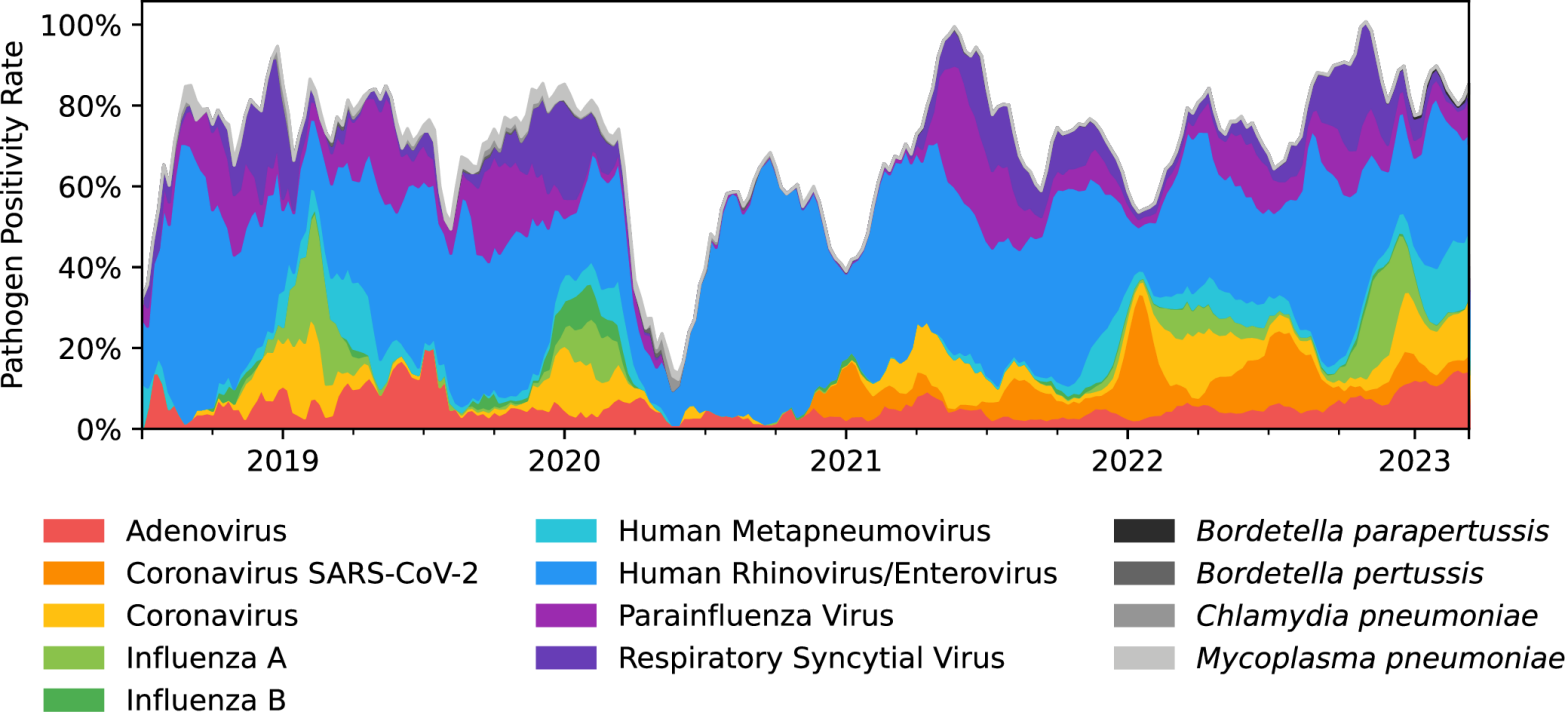
Overall positivity rate by season and year.

Following the onset of the COVID pandemic, pathogen positivity by season dropped significantly. This can be seen in the spring and summer of 2020 compared to prior years (Fig. 1 and 2) and during the winter season for the following 2 years. Comparing tests from 2018 to 2019 and from 2021 to 2023 (i.e., excluding 2020), we observe a statistically significant difference in positivity for winter (*P* < 0.001) while no statistical difference was observed for the other seasons, though fall 2022 had substantially higher positivity rates compared to any other year. Winter 2021 and winter 2022 had similar positivity rates to winter 2020. In the summer and fall of 2020, most viral pathogen detection rates were reduced to nearly 0%, with the exception of human rhinovirus/enterovirus, where the positivity rate observed was comparable to or higher than prior years reaching 41.8% and 55.7% overall test positivity rate in the summer and fall, respectively (Fig. 2).

Coinfections (tests that were positive for more than one pathogen) were observed in 3,788 tests. Of these, 3,414 had two pathogens detected and 374 had three pathogens detected. Almost all (99%) coinfections were viral-viral and there were no substantial differences in viral pathogen positivity pre- and during-pandemic (other than SARS-CoV-2). For viral-bacterial coinfections, only *Chlamydia pneumoniae* and *Mycoplasma pneumoniae* rates differed before and during the pandemic (other than *Bordetella parapertusis*; Table 2). No bacterial-bacterial coinfections were detected.

**TABLE 2:** Coinfection positivity before and during pandemic

	Before pandemic	During pandemic
Total	403 (9.2%)	3,385 (9.8%)
Any viral pathogen (% positivity)	403 (100.0)	3,385 (100.0)
Adenovirus	124 (30.8%)	1,124 (33.2%)
Coronavirus	100 (24.8%)	711 (21.0%)
Coronavirus SARS-CoV-2		485 (14.6%)
Human metapneumovirus	45 (11.2%)	413 (12.2%)
Human rhinovirus/enterovirus	295 (73.2%)	2,462 (72.7%)
Influenza A	32 (7.9%)	247 (7.3%)
Influenza B	15 (3.7%)	82 (2.4%)
Parainfluenza virus	97 (24.1%)	807 (23.8%)
Respiratory syncytial virus	106 (26.3%)	754 (22.3%)
Any bacterial pathogen	20 (5.0%)	31 (0.9%)
*Bordetella parapertussis*		16 (0.5%)
*Bordetella pertussis*	3 (0.7%)	8 (0.2%)
*Chlamydia pneumoniae*	3 (0.7%)	1 (0.03%)
*Mycoplasma pneumoniae*	14 (3.5%)	6 (0.2%)

To understand distribution by population density, we stratified sites into rural, suburban, and urban sites based on population density at the zip code level for each site. Differences in pathogen positivity rates by population density were heavily influenced by the inclusion of one urban site that made up 43% of test results. In sensitivity analyses when this site was not included, no differences in positivity rate between high, medium, and low population densities were observed.

## DISCUSSION

Our overarching objective was to understand the testing patterns and pathogen detection rates in pediatric outpatient facilities over 5 years using real-world data. By analyzing 38,778 unique test results from 16 pediatric facilities, the study presents several important insights into changes in respiratory pathogen dynamics during the COVID-19 pandemic, general pediatric respiratory pathogen epidemiology, and pediatric respiratory coinfection rates. The primary analyses revealed that the portion of tests in a year with detection of any pathogen fluctuated between 51.9% (2020) and 67.8% (2023), with the majority of detections indicating viral etiology reflecting the high rates of viral infections among pediatrics both before and after the onset of SARS-CoV-2 circulation.

Uncertainty in the circulation of pediatric viral illness after the COVID-19 pandemic disruption has been reported (11). Winter peaks of RSV and influenza, along with the seasonal variation of other circulating viruses, were disrupted with the onset of the COVID-19 pandemic, at least somewhat attributable to social distancing measures (11 – 13). When evaluating our data for seasonal variation in pathogen detections from 2018 to 2023, it is clear that in pediatric outpatient facilities, much of the heterogeneity of etiology of viral illness disappeared during the onset of the pandemic. In contrast, as shown in those data and the comparison of pre- and post-pandemic onset, the etiological heterogeneity of viral illness has been somewhat lower but overall maintained as time has continued. Similar surveillance trends and conclusions have been seen in the CDC’s NREVSS data for RSV, which recently reported a seasonal pattern and circulation for 2022–2023 that more closely resembled the pre-pandemic era (14). Additionally, of note, we found that a significant majority of samples with coinfections were viral-viral in nature. Viral-viral coinfections may mitigate ARTI symptoms among individuals due to viral competition (15). Conversely, viral-bacterial coinfections, which we found in a very small percentage of our study sample, are thought to present more symptomatically.

There are several antimicrobial stewardship implications of our study. As noted previously, with 30%–50% of outpatient ARTI prescribing being inappropriate and antibiotic use being the major driver of antimicrobial resistance, increased understanding of the circulating epidemiology among pediatric patients in the outpatient setting may facilitate improved prescribing practices, particularly with the data from the endemic COVID-19 era (2, 3). Diagnostic uncertainty is known to impact prescribing (16) along with other factors, such as local prescribing practices and provider care settings; for example, urgent care centers with high volumes of encounters have been associated with inappropriate prescribing (17). Routine, extensive multiplex PCR testing may decrease inappropriate prescribing in addition to providing a better understanding of pediatric respiratory epidemiology (18). However, such efforts need strong implementation considerations to ensure pre-analytical and post-analytical optimizations for maintaining the clinical utility of the testing (16, 18, 19). In addition to improving efforts in AMR, test-and-treat strategies are important to decrease inappropriate antibiotics to avoid adverse effects and increase the appropriateness of antiviral prescribing, which has been associated with decreased admissions and mortality among high-risk ambulatory patients (20 – 22).

There are several limitations to this study. The testing panels for each of the multiplex PCR tests used, while extensive, are not exhaustive for all circulating viruses (e.g., bocavirus) and additionally include a limited number of bacterial targets (23). Our study data were based on a convenience sample of sites, chosen based on the facility’s volume of testing and historical duration of testing, in addition to voluntary data contribution opt in by sites. Thus, our results may not be representative due to possible sampling bias. However, other voluntary surveillance approaches such as the CDC NREVSS have similar limitations in sampling (14). The diagnostic tests used did not include COVID-19 detection until 11 November 2020 (when RP2.1-EZ was put into use), so another limitation is our inability to describe COVID-19 positivity at the beginning of the pandemic [between March (WHO pandemic announcement on 10 March 2020) and November].

In both the pre-COVID-19 era and since the onset of SARS-CoV-2 circulation, variations in testing practices could have led to selection bias of specific patients for testing with extensive multiple PCR panels, which may lend to these results not being representative (12, 14). However, BIOFIRE Syndromic Trends, a software that provides local and regional pathogen circulation trends and aggregates data from highly multiplex PCR testing, has benchmarked well against other surveillance including CDC’s Foodnet and FluView (10, 24). This benchmarking suggests the possibility of a selection bias for highly multiplex PCR vs targeted testing methods does not limit the conclusions of our research. Future research may delve deeper into understanding testing pattern variations in practice and their implications for public health planning and disease prevention strategies in surveillance.

### Conclusion

This comprehensive analysis provides important insights into testing patterns, pathogen detection rates, and their temporal distribution in pediatric outpatient facilities over a span of 5 years. It reveals a notable fluctuation in the detection of any pathogen, largely dominated by viral etiologies. The data also suggest a shift in the heterogeneity of the etiology of viral illness following the onset of the COVID-19 pandemic that subsequently has returned to an epidemiologic pattern similar to the pre-pandemic era. Importantly, these findings highlight potential opportunities for improved isolation precautions and antimicrobial stewardship, particularly regarding the use of antibiotics and antivirals in outpatient settings. However, due to the possibility of sampling and selection bias, further research is needed to fully understand these trends and their implications for disease prevention strategies and public health planning.

## SUPPLEMENTAL MATERIAL

Supplemental material spectrum.03423-23-s0001.docx Tables S1 and S2.

ASM does not own the copyrights to Supplemental Material that may be linked to, or accessed through, an article. The authors have granted ASM a non-exclusive, world-wide license to publish the Supplemental Material files. Please contact the corresponding author directly for reuse.
