# Supplementary material for: The epidemiology of pediatric outpatient acute respiratory tract infections in the US: a multi-facility analysis of multiplex PCR testing from 2018 to 2023
Source: Microbiol Spectr. 2023 Dec 14;12(1):e03423-23. doi: 10.1128/spectrum.03423-23 (PMC10782947; doi:10.1128/spectrum.03423-23)
Supplement: Supplemental material — Tables S1 and S2. [file spectrum.03423-23-s0001.docx]

**Supplementary Materials**

**Table S1**. Number of Tests by Year, Region, and State

| **Year** | **Region** | **State** | **Number of Tests** |
| --- | --- | --- | --- |
| 2018 | Northeast | CT, MD | 376 |
|  | South | AL, LA | 296 |
| 2019 | Northeast | CT, MD | 701 |
|  | South | AL, LA, TX | 1476 |
|  | West | CA | 481 |
| 2020 | Northeast | CT, MD | 1582 |
|  | South | AL, FL, KY, LA, TX | 1986 |
|  | West | CA | 566 |
| 2021 | Northeast | CT, MD | 3173 |
|  | South | AL, KY, LA, TX | 4981 |
|  | West | CA | 6390 |
| 2022 | Northeast | CT, MD | 2068 |
|  | South | AL, KY, LA, TX | 3990 |
|  | West | CA | 8613 |
| 2023 | Northeast | CT | 76 |
|  | South | AL, KY, LA, TX | 648 |
|  | West | CA | 1375 |

**Table S2.** Overall positivity rate of pre- and during pandemic

|  | Before pandemic | During pandemic |
| --- | --- | --- |
| Total Tests  (% total tests) | 4399 (11.3%) | 34379 (88.7%) |
| Any Pathogen  (% positivity) | 2941 (66.9%) | 21107 (61.4%) |
| Any Viral Pathogen | 2870 (65.2%) | 21081 (61.3%) |
| Adenovirus | 273 (6.2%) | 1790 (5.2%) |
| Coronavirus | 282 (6.4%) | 1847 (5.4%) |
| Coronavirus SARS-CoV-2* |  | 2838 (8.8%) |
| Human Metapneumovirus | 199 (4.5%) | 1310 (3.8%) |
| Human Rhinovirus/Enterovirus | 1385 (31.5%) | 11095 (32.3%) |
| Influenza A | 227 (5.2%) | 1183 (3.4%) |
| Influenza B | 126 (2.9%) | 146 (0.4%) |
| Parainfluenza Virus | 406 (9.2%) | 2381 (6.9%) |
| Respiratory Syncytial Virus | 383 (8.7%) | 2191 (6.4%) |
| Any Bacterial Pathogen | 91 (2.1%) | 57 (0.2%) |
| *Bordetella parapertussis** |  | 22 (0.07%) |
| *Bordetella pertussis* | 6 (0.1%) | 13 (0.04%) |
| *Chlamydia pneumoniae* | 19 (0.4%) | 8 (0.02%) |
| *Mycoplasma pneumoniae* | 66 (1.5%) | 14 (0.04%) |

*RP2.1-EZ test only
